# Developmental Toxicity of Fine Particulate Matter: Multifaceted Exploration from Epidemiological and Laboratory Perspectives

**DOI:** 10.3390/toxics12040274

**Published:** 2024-04-06

**Authors:** Ruifeng Yan, Danni Ma, Yutong Liu, Rui Wang, Lifan Fan, Qiqi Yan, Chen Chen, Wenhao Wang, Zhihua Ren, Tingting Ku, Xia Ning, Nan Sang

**Affiliations:** College of Environment and Resource, Research Center of Environment and Health, Shanxi University, Taiyuan 030006, China; yanruifeng@sxu.edu.cn (R.Y.); 18535704463@163.com (D.M.); 202313905002@email.sxu.edu.cn (Y.L.); wir010202@163.com (R.W.); 202223905001@email.sxu.edu.cn (L.F.); yqq13134680242@163.com (Q.Y.); 13347541630@163.com (C.C.); wwh11232002@163.com (W.W.); zhren@sxu.edu.cn (Z.R.); ningxia@sxu.edu.cn (X.N.); sangnan@sxu.edu.cn (N.S.)

**Keywords:** fine particulate matter, adverse birth outcomes, respiratory development, cardiovascular development, neurological development, mechanism

## Abstract

Particulate matter of size ≤ 2.5 μm (PM_2.5_) is a critical environmental threat that considerably contributes to the global disease burden. However, accompanied by the rapid research progress in this field, the existing research on developmental toxicity is still constrained by limited data sources, varying quality, and insufficient in-depth mechanistic analysis. This review includes the currently available epidemiological and laboratory evidence and comprehensively characterizes the adverse effects of PM_2.5_ on developing individuals in different regions and various pollution sources. In addition, this review explores the effect of PM_2.5_ exposure to individuals of different ethnicities, genders, and socioeconomic levels on adverse birth outcomes and cardiopulmonary and neurological development. Furthermore, the molecular mechanisms involved in the adverse health effects of PM_2.5_ primarily encompass transcriptional and translational regulation, oxidative stress, inflammatory response, and epigenetic modulation. The primary findings and novel perspectives regarding the association between public health and PM_2.5_ were examined, highlighting the need for future studies to explore its sources, composition, and sex-specific effects. Additionally, further research is required to delve deeper into the more intricate underlying mechanisms to effectively prevent or mitigate the harmful effects of air pollution on human health.

## 1. Introduction

Particulate matter of size ≤ 2.5 μm (PM_2.5_) is defined as a type of fine PM with a diameter of <2.5 μm, which is distinguished by small particle dimensions, large relative surface area, and a capacity to adsorb heavy metals and toxic organic pollutants. These characteristics facilitate the infiltration of PM_2.5_ into the human body, leading to various adverse reactions. Extensive research has consistently demonstrated the harmful health effects of prolonged PM_2.5_ exposure, which predisposes individuals to an augmented risk of cardiovascular and respiratory ailments as well as neurological disorders such as Alzheimer’s disease [1,2,3]. Moreover, outdoor air pollution, specifically that caused by ambient PM, has been officially classified as a Class I human carcinogen by the International Agency for Research on Cancer [4].

During early life, which is a pivotal stage of individual development, the body’s system exhibits heightened sensitivity to environmental pollutants and is more prone to their toxic effects compared with adults. Therefore, there is a substantial interest in exploring the potential causal link between PM_2.5_ exposure in early life and a wide array of diseases. An increasing amount of epidemiological research has established a correlation between developmental exposure to PM_2.5_ and the heightened risk of birth defects and various diseases [5,6,7,8]. PM_2.5_ exposure during pregnancy can lead to negative consequences such as preterm labor (PTB), low birth weight (LBW), and multiple facial defects [9,10,11]. Furthermore, prenatal and postnatal exposures to PM_2.5_ reportedly impact lung development [12,13], heighten vulnerability to respiratory infections, and potentially contribute to the occurrence of respiratory ailments during early childhood [12]. The high level of maternal exposure to air pollution is also correlated with the increased chances of congenital heart defects (CHDs) in the offspring and has a detrimental effect on their neurodevelopment in early childhood [14,15,16]. Additionally, even minimal levels of air pollution exposure during pregnancy (levels that might not significantly affect adults) reportedly disrupt fetal development and result in long-term dysfunction [17].

Considering the escalating public concern regarding the health hazards associated with PM_2.5_ during early life, the effects of prenatal exposure to PM_2.5_ on fetal health remain uncertain. This review systematically investigates the detrimental biological effects of these pollutants on the developmental outcomes of humans by incorporating approximately 20 years of epidemiological and laboratory evidence, encompassing the latest research findings and advancements (epidemiological evidence is presented in Table 1). This review emphasizes the differences in the developmental toxicity of PM_2.5_ from different regions and pollution sources as well as variations among different ethnicities, sexes, and socioeconomic levels, through the comprehensive analysis and comparison of various research outcomes.

Integrating and summarizing the commonalities found in these data reveal the complexity and diversity of PM_2.5_ developmental toxicity, which can provide readers with a comprehensive background and foundation for better understanding the current status and trends in this field. Concurrently, this study comprehensively elucidates the possible molecular mechanisms underlying the health impairments triggered by PM_2.5_ in various systems, mainly involving transcriptional and translational regulation, oxidative stress and inflammatory responses, and epigenetic regulation [18], providing deeper theoretical support and guidance for research in related fields. The primary conclusions and novel perspectives regarding the relation between public health and PM_2.5_ are summarized in this review, which might offer insights into the prevention and treatment of environment-related illnesses, assist with the development of clinically applicable drugs, and serve as specific instructions for future research directions.

**Table 1:** Epidemiological studies on the adverse developmental outcomes of prenatal or postnatal PM_2.5_ exposure.

Country	Contaminant	Exposure Period	Outcome	Supporting Statistics	References
China	PM_2.5_	During the entire pregnancy	PROM	A substantial link between acute PM_2.5_ exposure (per interquartile range increase) and the likelihood of PROM (OR = 1.11; 95% CI: 1.03, 1.19 for PM_2.5_ on delivery day; OR = 1.10; 95% CI: 1.02, 1.18 for PM_2.5_ 1 day before delivery)	Wang et al., 2022 [19]
USA	PM_2.5_	During the entire pregnancy	PTB	High levels of PM_2.5_ exposure were correlated with a 19% increase in PTB risk	Defranco et al., 2016 [20]
China	PM_2.5_ and PM_10_	During the entire pregnancy	PTB and near-term birth	Exposure to PM_2.5_ or PM_10_ throughout pregnancy increased the likelihood of PTB and near-term birth (HR: 1.09 [95% CI: 1.08, 1.10] for a 10 μg/m^3^ increase in PM_2.5_)	Li et al., 2018 [21]
USA	PM_2.5_ and NO_2_	During the entire pregnancy	Declined birth weights	For each 10 µg/m^3^ increase in PM_2.5_ exposure, birth weights underwent a decline of 18.4, 10.5, 29.7, and 48.4 g during the first, second, and third trimesters, or throughout the entire pregnancy, respectively	Savitz et al., 2014 [22]
USA	PM_2.5_	During the entire pregnancy	LBW	A mean concentration increase in PM_2.5_ exposure was linked to a 3.2% (95% CI = −1.0%, 6.3%) increase in the probability of LBW among term births	Kirwa et al., 2019 [23]
Republic of Chile	PM_2.5_ and PM_10_	Gestational exposure	LBW	PM_2.5_ exposure during the second trimester was associated with a higher likelihood of LBW (OR: 1.031; 95% CI: 1.004–1.059)	Rodríguez-Fernández et al., 2022 [24]
USA	PM_2.5_, PM_10_, CO	During 3 months prior to conception and gestational weeks 3–8	Orofacial defects, CP	The positive associations of PM_2.5_ with CP were most eminent from gestational weeks 3–5	Zhu et al., 2015 [5]
USA	PM_2.5_ and O_3_	During early gestation (weeks 5–10 of gestation)	CP	Each 10 μg/m^3^ increase in PM_2.5_ concentration was accompanied by a 43% increase in the risk of developing CP	Zhou et al., 2017 [25]
China	PM_2.5_	Elementary school students aged 5–12	Impaired respiratory health, increased airway inflammation, reduced lung function	Among all constituents of PM_2.5_, organic carbon, elemental carbon, NO_3_^−^, and NH_4_^+^ had the consistent and strongest associations with airway inflammation biomarkers and lung function parameters, followed by metallic elements	Wu et al., 2021 [26]
Poland	PM_2.5_	During the entire pregnancy	Increased susceptibility to respiratory infections in early childhood	The aOR for the occurrence of recurrent bronchopulmonary infections during the follow-up period significantly increased in a dose–response relationship with the prenatal PM_2.5_ level (OR = 2.44, 95% CI: 1.12–5.36)	Jedrychowski et al., 2013 [12]
China	PM_2.5_	During pregnancy and infancy	Asthma	The susceptible periods of developing asthma included gestational weeks 6–22 and 9–46 weeks following delivery	Jung et al., 2019 [27]
USA	PM_2.5_	During the entire pregnancy	Asthma	Boys with higher prenatal exposure during midgestation (16–25-week gestation) exhibited an increased incidence of developing asthma by the age of 6 years	Hsu et al., 2015 [28]
USA	PM_2.5_	During established morphogenic phases of prenatal lung development	Asthma	An increase in PM_2.5_ by 2 μg/m^3^ was found to have a connection with a 1.29-fold higher risk of developing asthma	Hazlehurst et al., 2021 [29]
USA	PM_2.5_	Potential	Repeated wheezing	Heightened prenatal exposure to PM_2.5_ during the later stages of pregnancy correlated with recurrent wheezing in early childhood	Chiu et al., 2022 [30]
Israeli	CO, NO_2_, O_3_, SO_2_, PM_10_, PM_2.5_	During weeks 3–8 of pregnancy	Multiple CHDs	Higher maternal exposure to PM_10_ was linked to an elevated likelihood of multiple CHDs (aOR 1.05, 95% CI: 1.01–1.10 for a 10 μg/m^3^ increment)	Agay-Shay et al., 2013 [31]
China	PM_10_, PM_2.5_, NO_2_, CO, SO_2_	During the entire pregnancy	CHDs	Markedly increasing the association of PM_2.5_ exposure during the second and third trimesters with the occurrence of CHDs, with aORs of 1.228 and 1.236 (95% CI: 1.141–1.322, 1.154–1.324 individually) for each 10 μg/m^3^ increase in PM_2.5_ level	Sun et al., 2022 [16]
USA	Benzene and PM_2.5_	During gestation	Selected heart defects	Exposure to elevated concentrations of PM_2.5_ was linked to a higher risk of developing specific heart defects, including truncus arteriosus, the coarctation of the aorta, and interrupted aortic arch	Tanner et al., 2015 [32]
USA	PM_2.5_	During pregnancy	Increased BP in children aged 3–9 years old	A 5 μg/m^3^ rise in PM_2.5_ during the third trimester was associated with a 3.49 percentile (95% CI: 0.71–6.26) elevation in child SBP or a 1.47 times (95% CI: 1.17–1.85) higher hazard of elevated BP	Zhang et al., 2018 [9]
USA	PM_2.5_ and BC	Each trimester of pregnancy and within 2–90 days before birth	Elevated BP among newborns	Exposure to PM_2.5_ and BC during the late pregnancy stage was associated with elevated BP among newborns (e.g., 1.0 mmHg; 95% CI: 0.1, 1.8 for a 0.32 μg/m^3^ increase in mean 90-day residential BC)	Van Rossem et al., 2015 [33]
Spain	PM_2.5_ and NO_2_	Throughout the whole period of the pregnancies	Adverse impacts on memory and verbal abilities in boys	Boys being more susceptible between the ages of 4 and 6 years, particularly in areas associated with memory, verbal aptitude, and overall cognitive abilities	Lertxundi et al., 2019 [34]
USA	PM_2.5_	Entire pregnancy	ID	The risks of ID associated with having daily average PM_2.5_ concentrations over the current US NAAQS threshold (i.e., ≥12.0 μg/m^3^) during the preconception and first trimester windows were substantial; the odds ratios were 1.8 and 2.4, respectively	Grineski et al., 2023 [35]
USA	PM_2.5_	Prenatal	Slightly lower IQ in late childhood	A substantial correlation was observed between PM_2.5_ exposure in the later stages of pregnancy (months 5–7) and the IQ of children	Holm et al., 2023 [36]
China	PM_2.5_ and PM_10_	Prenatal and early postnatal exposures	Decreased MDI and PDI scores	Exposure to PM_2.5_ and PM_10_ in either of the two periods was linked to reduced scores of the MDI and PDI in the offspring	Wang et al., 2022 [14]
China	PM_2.5_	Prenatal	SDDs	PM_2.5_ exposure may elevate the risk of SDDs in both sexes (RR: 1.52, 95% CI: 1.19, 2.03, per 10 μg/m^3^ increase in PM_2.5_ exposure), particularly in problem-solving skills among girls (RR: 2.23, 95% CI: 1.22, 4.35)	Wang et al., 2021 [37]
China	PM_2.5_	Prenatal and postnatal	Decreased LDCDQ scores and increased risk of DCD	Exposure to PM_2.5_ was linked to diminished motor performance and a greater risk of DCD, with an aOR of 1.06 (95% CI: 1.01, 1.10) and 1.06 (95% CI: 1.01, 1.13) for every interquartile range elevation in PM_2.5_ exposure during the first 3 months and the initial 3 years, separately	Cai et al., 2023 [38]
China	PM_2.5_	During the prenatal and postnatal periods	Postponement in gross motor, fine motor, and personal–social development	Exposure during the second trimester of pregnancy was linked to an elevated probability of delayed neurodevelopment related to gross motor skills (aOR: 1.09 per 10 μg/m^3^ increase). The delayed development of fine motor skills was revealed to be involved with PM_2.5_ exposure in the second and third trimesters (aOR: 1.06)	Shih et al., 2023 [39]
USA	PM_2.5_	From 3 months before pregnancy until the child’s second birthday	ASD	A 50% rise in the risk of ASD when exposed to an average cumulative level of PM_2.5_ from 3 months before conception through the child’s second year (*p* = 0.046)	Talbott et al., 2015 [40]
USA	PM_2.5_ and PM_10_	From 9 months before pregnancy to 9 months after delivery	Greater odds of ASD	The relation between ASD and PM_2.5_ exposure was more pronounced in the final trimester (OR = 1.42 per IQR raise in PM_2.5_; 95% CI: 1.09, 1.86)	Raz et al., 2015 [41]
USA	PM_2.5_, NO_2_, O_3_	During pregnancy	ASD	The sensitive periods of PM_2.5_ exposure linked to ASD occurred early in pregnancy, demonstrating statistical significance over 1–27 weeks of gestation (cumulative HR = 1.14 [95% CI: 1.06, 1.23] per IQR [7.4 μg/m^3^] increase)	Rahman et al., 2022 [42]
USA	Aircraft ultrafine particles	During pregnancy	ASD	A significant association was identified between the increased risk of ASD and maternal exposure to aircraft PM_0.1_ throughout pregnancy (HR: 1.02, [95% CI: 1.01–1.03] per interquartile range [IQR] = 0.02 μg/m^3^ rise)	Carter et al., 2023 [43]

Abbreviations: ASD, autism spectrum disorders; BC, black carbon; BP, blood pressure; CHDs, congenital heart diseases; CP, cleft palate alone; DCD, developmental coordination disorder; ID, intellectual disability; IQ, Intelligence Quotient; LDCDQ, Little DCD Questionnaire; MDI, Mental Developmental Index; PDI, Psychomotor Developmental Index; PROM, premature rupture of membranes; and SDD, suspected developmental delay.

## 2. Materials and Methods

### 2.1. Search Strategy

We performed a literature review using the PubMed database to identify articles published in the last 20 years (2003–2023) regarding the developmental toxicity of PM_2.5_ exposure. We utilized various combinations of the following keywords: “air pollution” or “particulate matter” or “PM” and “pregnancy” or “child” or “offspring” or “developmental toxicity”. Additionally, the studies identified through the aforementioned search strategy were examined.

### 2.2. Inclusion Criteria

Studies were included if they involved (1) epidemiological or clinical investigations evaluating the effect of maternal exposure to PM_2.5_ on offspring development, exploring the association between PM_2.5_ and various adverse outcomes such as neurological disorders and cardiopulmonary diseases in offspring; (2) research delving into the developmental toxicity and underlying mechanisms of PM_2.5_ utilizing in vivo and in vitro models with commonly utilized laboratory materials (e.g., mice, rats, and zebrafish); or (3) comprehensive analysis of the mechanisms underlying developmental toxicity induced by PM_2.5_, encompassing aspects such as transcriptional and translational regulation, oxidative stress, inflammatory responses, epigenetic modifications, and other pertinent factors.

### 2.3. Exclusion Criteria

The studies were excluded if they were (1) not written in English, (2) published before 2003, (3) if their full text was not available from PubMed, or (4) if they addressed irrelevant populations or exposures not during the developmental period.

## 3. Adverse Birth Outcomes Induced by PM_2.5_

### 3.1. Epidemiological Studies

#### 3.1.1. Preterm Birth

The present findings confirmed that maternal exposure to PM_2.5_ was correlated with an increased probability of preterm labor. A nationwide survey in China revealed that maternal PM_2.5_ exposure, whether acute or chronic, considerably heightened the likelihood of PROM, resulting in a heightened risk of negative outcomes for mothers and newborns, including preterm birth, intrauterine infection, and abruptio placentae [19]. A substantial link has been reported between acute PM_2.5_ exposure (per interquartile range increase) and the likelihood of PROM (odds ratio [OR] = 1.11; 95% confidence interval [CI]: 1.03, 1.19 for PM_2.5_ on delivery day; OR = 1.10; 95% CI: 1.02, 1.18 for PM_2.5_ one day before delivery).

Women with lower levels of education or obesity or who gave birth during colder seasons appeared to be more sensitive to the effects of ambient PM_2.5_ [19]. In a cohort study conducted in Ohio, high levels of PM_2.5_ exposure during pregnancy were correlated with a 19% increase in PTB risk, with the highest risk occurring during late pregnancy [20]. This study also employed a logistic regression model to identify other factors linked to preterm birth, notably maternal race. After accounting for other variables, non-Hispanic black mothers demonstrated a heightened risk of preterm birth (aOR 1.46) compared to non-Hispanic white mothers (aOR 1.00), with a statistically significant association. Moreover, a cohort comprising >1.28 million births in China demonstrated that exposure to PM_2.5_ or PM_10_ throughout pregnancy increased the likelihood of PTB and near-term birth (hazard ratio [HR]: 1.09 [95% CI: 1.08, 1.10] for a 10 μg/m^3^ increase in PM_2.5_). Women with poor socioeconomic backgrounds, obesity, and smoking habits were more susceptible to PM exposure [21]. Taken together, these findings highlight the significance of considering the actual background of pregnant women, such as income levels and racial differences, in analyzing the toxic effects of PM_2.5_.

#### 3.1.2. Low Birth Weight and Reduced Fetal Growth

Birth weight is a well-known predictor of overall neonatal health. There is a well-established link between LBW and elevated neonatal mortality and morbidity rates [7]. In recent years, researchers have accumulated evidence indicating a direct association between increased prenatal exposure to PM_2.5_ and higher probability of LBW. A study including 252,967 births conducted from 2008 to 2010 reported that for each 10 µg/m^3^ increase in PM_2.5_ exposure, birth weights underwent a decline of 18.4, 10.5, 29.7, and 48.4 g during the first, second, and third trimesters, or throughout the entire pregnancy, respectively [22]. An extensive analysis involving >330,000 births to Hispanic and Black mothers spanning 14 years in Puerto Rico noted that a mean concentration increase in PM_2.5_ exposure was linked to a 3.2% (95% CI = −1.0%, 6.3%) increase in the probability of LBW among term births [23].

A cross-sectional analytical project comprising 595,369 Chilean newborns investigated the connection between LBW and gestational exposure to PM_2.5_ and PM_10_. The investigation concluded that PM_2.5_ exposure during the second trimester was associated with a higher likelihood of LBW (OR: 1.031; 95% CI: 1.004–1.059), whereas PM_10_ had an impact on the entire duration of pregnancy [24]. These findings strongly support the correlation between urban air pollution exposure and LBW as well as diminished fetal growth.

#### 3.1.3. Facial Defects

While there is supporting evidence demonstrating a correlation between maternal ambient air pollution exposure and the occurrence of congenital orofacial clefts in children, epidemiologic research has produced inconclusive findings. A large retrospective cohort in the US investigated substantial and positive relevance between ambient air pollution and the risk of orofacial defects in the offspring of mothers who were exposed to PM_2.5_ during the 3 months before conception [5]. Specifically, the positive associations of PM_2.5_ with cleft palate were most eminent from gestational weeks 3–5 when analyzed in terms of individual week [5]. According to a comparable study, each 10 μg/m^3^ increase in PM_2.5_ concentration was accompanied by a 43% increase in the risk of developing cleft palate [25]. Conversely, no substantial correlation was detected between the concentration of PM_2.5_ and the incidence of cleft lip [25], which also indicates potential uncertainty regarding facial defects in relation to PM_2.5_ exposure. In addition to facial defects, it is crucial to further explore other types of birth defects associated with PM_2.5_ exposure to address existing gaps in knowledge.

### 3.2. Experimental Research

Experimental studies demonstrated that PM_2.5_ exposure inhibited embryonic development, which aligned with the aforementioned epidemiological observations. Offspring birth weight was substantially reduced owing to maternal exposure to concentrated ambient PM_2.5_ (CAP), while the body weight of adult male descendants showed an increase [44]. Similarly, a substantial decrease in body weight and crown-rump length on GD13 (gestation day) and GD18 was revealed in another study that evaluated fetal and placenta development exposure in utero in mice, which was concomitant with abnormal placental structure and aberrant placental functional gene expression [45]. The detrimental effects of PM_2.5_ on birth outcomes have also been extensively investigated through numerous zebrafish experiments. PM_2.5_ exposure caused developmental toxicity that varied with dose and duration [46], leading to increased mortality and malformations as well as decreased hatchability rate and body length [47]. These findings underscore the potential association between PM_2.5_ exposure during pregnancy and embryo growth restriction. However, as experimental model organisms, mice exhibit differences from humans or other animals in aspects such as physiological structure, metabolic pathways, and behavioral patterns, making it inappropriate to directly extrapolate findings to other species or natural ecosystems. Subsequent epidemiological and toxicological investigations are required to examine the consequences of PM_2.5_ on other species.

## 4. Adverse Effects of PM_2.5_ on Respiratory Development

### 4.1. Epidemiological Studies

The development of lungs is a complex process that spans from conception to the postnatal period [27]; particulate air pollution impacts lung development during the prenatal and postnatal period [12,13], increases the likelihood of contracting respiratory infections, and could cause respiratory health issues during early childhood [12]. An increasing number of studies have associated PM_2.5_ exposure with children’s respiratory health, with findings revealing that developmental exposure can disrupt alveolarization, compromise lung function, and alter pulmonary immune differentiation, potentially impacting short- and long-term health conditions [48,49]. According to a longitudinal panel investigation with repeated health assessments conducted in Shanghai involving 62 children, short-term PM_2.5_ exposure can compromise children’s respiratory health, increasing airway inflammation, reducing lung function, and altering the microbial colonization of the buccal mucosa [26]. In a separate study involving 214 children monitored over 7 years, the adjusted OR (aOR) for the occurrence of recurrent bronchopulmonary infections during the follow-up period significantly increased in a dose–response relationship with the prenatal PM_2.5_ level (OR = 2.44, 95% CI: 1.12–5.36) [12]. This study also reported that setting the 24 h average PM_2.5_ concentration at 20 μg/m^3^ as a target value can offer superior protection for unborn babies compared with the previously established EPA guidelines.

In addition, clinical respiratory symptoms of particulate pollution aggravate asthma [50]. According to a large birth cohort study conducted in Taichung City, there is a notable association between augmented prenatal and postnatal exposure to PM_2.5_ and an elevated risk of developing asthma. The susceptible periods included gestational weeks 6–22 and 9–46 weeks following delivery [27]. Boys with higher prenatal exposure during midgestation (16–25-week gestation) exhibited an increased incidence of developing asthma by the age of 6 years [28]. In a combined analysis of two pregnancy cohorts, children living with higher PM_2.5_ concentrations during the saccular stage of fetal lung development were more likely to develop asthma. An increase in PM_2.5_ by 2 μg/m^3^ was found to have a connection with a 1.29-fold higher risk of developing asthma [29]. The potential impact of PM_2.5_ on offspring may exhibit sex-specific and time-dependent consequences, which appear to differ based on ethnicity and level of maternal antioxidant intake. Research on a multiethnic inner-city population revealed that heightened prenatal exposure to PM_2.5_ during the later stages of pregnancy correlated with recurrent wheezing in early childhood, specifically among male infants born to Black mothers with low levels of antioxidant consumption [30]. Existing research has provided valuable insights into the association between PM_2.5_ exposure and respiratory health by considering gender and racial differences.

### 4.2. Experimental Research

Animal studies revealed that maternal exposure to PM_2.5_ during the sensitive windows resulted in fetal pulmonary dysfunction and increased the risk of lung diseases. In a rat model, prolonged exposure during pregnancy substantially modified the structure and function of the lungs in the offspring [51], characterized by heightened lung consolidation, airway inflammation, and diminished lung volume and compliance [52]. Another study reported various changes, such as interstitial proliferation in the lungs, notable oxidative stress, and enhancement of epithelial–mesenchymal transition (EMT) [53]. Moreover, previous findings revealed that male descendants experienced a dysplasia-like syndrome, encompassing hypoalveolarization, diminished secretory function, delayed microvascular development, and inflammation within the lungs. Nonetheless, female offspring exhibited only slight changes in alveolarization and lung inflammation, indicating that male offspring were more vulnerable to maternal PM_2.5_ exposure. Interestingly, these negative effects were nearly reversed during postnatal development [54]. There are physiological, immunological, and metabolic differences between genders that may result in divergent responses to PM_2.5_ exposure. However, current research predominantly focuses on the general population, without fully considering the impact of gender differences on susceptibility to PM_2.5_. There is a lack of comprehensive understanding regarding the gender-specific respiratory diseases of PM_2.5_.

## 5. Cardiovascular Diseases Induced by PM_2.5_

### 5.1. Epidemiological Studies

#### 5.1.1. Heart Defects

There is considerable evidence suggesting that maternal exposure to air pollution is positively correlated with the risk of CHD in offspring [55,56]. A cohort study based on registries employed a spatiotemporal approach using Geographic Information System data and weekly inverse distance weighting modeling to examine the potential connections between exposure to ambient air pollution during week 3–8 of pregnancy and the risk of CHD. The results indicated that higher maternal exposure to PM_10_ was linked to an elevated likelihood of multiple CHD (aOR 1.05, 95% CI: 1.01–1.10 for a 10 μg/m^3^ increment), while higher exposure to PM_2.5_ was linked to a reduced risk for patent ductus arteriosus (aOR 0.78, 95% CI: 0.68–0.91 for a 5-µg/m^3^ increment), and sensitivity analyses confirmed the consistency of these results [31].

The markedly increasing association of PM_2.5_ exposure during the second and third trimesters with the occurrence of CHDs was detected in a study in Suzhou, with aORs of 1.228 and 1.236 (95% CI: 1.141–1.322, 1.154–1.324 individually) for each 10 μg/m^3^ increase in PM_2.5_ level [16]. In a retrospective cohort study in Florida [32], exposure to elevated concentrations of PM_2.5_ was linked to a higher risk of developing specific heart defects, including truncus arteriosus, coarctation of the aorta, and interrupted aortic arch. However, it is currently uncertain whether these connections have meaningful clinical relevance or can be directly attributed to exposure to air pollution. The latter findings need to be supplemented by other studies for definitive conclusions.

#### 5.1.2. High Blood Pressure

Relevant evidence showed that air pollution exposure during pregnancy may also render offspring increasingly susceptible to high BP. A cohort study involving 1131 mother–infant pairs in Boston examined the potential correlation between systolic blood pressure (SBP) and air pollutant exposures using mixed-effects models, reporting that exposure to PM_2.5_ and BC during the late pregnancy stage was associated with elevated BP among newborns (e.g., 1.0 mmHg; 95% CI: 0.1, 1.8 for a 0.32 μg/m^3^ increase in mean 90 day residential BC) [33].

In another study, connections between maternal exposure to ambient PM_2.5_ and elevated child BP at 3–9 years old were detected in a cohort from the USA, suggesting that reducing maternal exposure could prevent pediatric hypertension at the primary level [9]. A 5 μg/m^3^ rise in PM_2.5_ during the third trimester was associated with a 3.49 percent (95% CI: 0.71–6.26) elevation in child SBP or a 1.47 times (95% CI:1.17–1.85) higher hazard of elevated BP, and the connection was partially explained by the impact of PM_2.5_ on fetal growth and weight during childhood, revealing novel perspectives of the mechanisms through which prenatal PM_2.5_ exposure influences SBP in children.

### 5.2. Experimental Research

Epidemiological evidence has shown that PM_2.5_ pollution during pregnancy may have a negative impact on cardiovascular system development. In fact, experimental research has corroborated the findings observed in humans.

Studies in mice revealed that PM_2.5_ exposure during the in utero period may affect the development of the descendant’s cardiovascular system and elevate the risk of altered BP [57,58,59] as well as contribute to heart failure and additional cardiovascular incidents [9,33,58]. Prominent histological changes were observed in the hearts of mature progeny mice exposed to PM_2.5_ pollution during gestation [60], primarily comprising disordered cardiac cell organization, inflammation, and enlarged myocardial septum. Furthermore, male mice exhibited more severe heart damage compared with female mice in mature progeny mice. A study presented compelling evidence that exposing gravida to PM_2.5_ at a concentration of 73.61 μg/m^3^ substantially increased cardiac dysfunction among male descendants during their adult years [57,58], which was evidenced by changes such as remodeling and dysfunction of the left ventricle in living organisms as well as dysfunction of cardiomyocytes in a laboratory setting. Pregnant mice exposed to diesel exhaust demonstrated an enhanced vulnerability to heart failure induced by pressure overload in the offspring as they matured into adulthood [61,62]. Cardiomyocytes extracted from mice exposed to PM_2.5_ during perinatal development also displayed changes in sarcomere function that were apparent at the cellular level [63].

Although these experiments were conducted solely in mice, similar observations of remarkable cardiovascular impairment caused by PM_2.5_ exposure are also present in other animal species. Specifically, several studies have documented detrimental impacts on the embryonic cardiac development of zebrafish [64]. TSP and PM_2.5_ exposure have been demonstrated to impair heart rate and blood flow velocity as well as affect cardiac morphology and angiogenesis [47]. Additional research substantiated that extractable organic matter (EOM) of PM_2.5_ was the primary cause underlying increased cardiac malformations, such as globular chambers and pericardial edema as well as decreased heart rate [64]. Moreover, it induced DNA damage and apoptosis, inhibited cardiac differentiation, and ultimately led to heart defects in zebrafish embryos and mouse P19 embryonic cancer cells [65,66,67]. Current research on the impact of PM_2.5_ on cardiovascular function is subject to certain limitations. Existing studies primarily focus on overall impacts while paying less attention to the specific components of PM_2.5_ responsible for the related biological processes. Additionally, there are limitations in research methods and techniques, such as a lack of high-resolution molecular analysis technology and research tools at the cellular level. Therefore, it is necessary for subsequent research to employ more advanced technological approaches to enhance the understanding of the precise constituents of PM_2.5_ that contribute to cardiovascular dysfunction and the related biological processes at the molecular level.

## 6. Neurodevelopmental Toxicity Caused by PM_2.5_

### 6.1. Epidemiological Studies

Exposure to PM_2.5_ and PM_10_ in the prenatal and early postnatal stages was found to have a detrimental impact on early childhood neurodevelopment [14,15].

#### 6.1.1. Developmental Delay and Developmental Coordination Disorder

According to relevant studies, preschoolers exposed to higher levels of PM_2.5_ exhibited higher levels of compromised neurobehavioral development, including developmental delay and DCD. Research involving the Shanghai Maternal–Child Pairs Cohort, which used the Ages and Stages Questionnaire (ASQ) to evaluate the neurological development of children at 2, 6, 12, and 24 months old, proposed that exposure to PM_2.5_ during weeks 18–34 of pregnancy showed a significant correlation with ASQ scores and SDDs. PM_2.5_ exposure may elevate the risk of SDDs in both sexes (RR: 1.52, 95% CI: 1.19, 2.03, per 10 μg/m^3^ increase in PM_2.5_ exposure), particularly in problem-solving skills among girls (RR: 2.23, 95% CI: 1.22, 4.35) [37]. Information gathered from a cohort study including 109,731 children between the ages of 3 and 5 years in China suggested that exposure to PM_2.5_ during prenatal and postnatal stages was linked to diminished motor performance and greater risk of DCD [38]. The aOR was 1.06 (95% CI: 1.01, 1.10) and 1.06 (95% CI: 1.01, 1.13) for every interquartile range elevation in PM_2.5_ exposure during the first 3 months and the initial 3 years, separately. The present investigation also suggested that infants who breastfed for <6 months exhibit a greater susceptibility to the effects of postnatal PM_2.5_ exposure.

In a cohort study conducted within the population of Taiwan, a notable correlation was observed between gestational exposure to PM_2.5_ and postponement in the development of gross motor skills, fine motor skills, and personal–social abilities [39]. Specifically, exposure during the second trimester of pregnancy was linked to an elevated probability of delayed neurodevelopment related to gross motor skills (aOR 1.09 per 10 μg/m^3^ increase). The delayed development of fine motor skills was revealed to be associated with PM_2.5_ exposure in the second and third trimesters (aOR 1.06), and personal–social skills were affected similarly. Nevertheless, the neurodevelopmental indicators mentioned above showed no correlation with postnatal PM_2.5_ exposure. Additional research examining the consequences of PM_2.5_ during pregnancy for neurodevelopment in children is essential.

#### 6.1.2. Cognitive Impairment

Cognition is the process by which organisms perceive and acquire knowledge, while cognitive impairment refers to the presence of difficulties in higher cortical functions such as perception, attention, language, memory, and thinking [68]. There is increasing evidence stating that prenatal or postnatal PM_2.5_ pollution affects the cognition of offspring. A case–control study in Utah (*n* = 1032) and a cohort study in California’s agricultural Salinas Valley (*n* = 568) concluded that encountering slightly elevated outdoor PM_2.5_ concentrations before delivery was indicative of greater odds of ID and small decrements in IQ in late childhood, which remained consistent across various sensitivity analyses [35,36].

According to a three-district birth cohort study carried out in Spain, the effects of PM_2.5_ exposure during pregnancy on neuropsychological development were observed to be dependent on the sex of the child, with boys being more susceptible between the ages of 4 and 6 years, particularly in areas associated with memory, verbal aptitude, and overall cognitive abilities [34]. Despite the potential cognitive benefits associated with breastfeeding, this study has revealed that exposure to environmental pollutants during intrauterine development may not be mitigated by breastfeeding. A substantial correlation was observed between PM_2.5_ exposure in the later stages of pregnancy (months 5–7) and the IQ of children, indicating that this specific time frame was particularly influential [36].

An interesting finding was the potential influence of the fetal sex on the PM_2.5_–IQ association. Boys exhibited low verbal comprehension and working memory with average PM_2.5_ exposure in utero, whereas girls exhibited a low processing speed. The connections between PM exposure during pregnancy or the early postnatal period and the neurodevelopment of offspring at 2 years of age were evaluated and compared by a study including 1331 mother–child pairs; the results indicated that exposure to PM_2.5_ and PM_10_ in either of the two periods was linked to reduced scores in the mental and psychomotor developmental indices in the offspring [14]. Additionally, compared with prenatal exposure, exposure during the early infancy phase more strongly affects neurodevelopment [14], indicating that the early postnatal phase may serve as a critical period for the impact of ambient PM on offspring development. Additional investigation is warranted to pinpoint the precise developmental timeframe when exposure to PM_2.5_ pollution has the most pronounced impact.

#### 6.1.3. Autism Spectrum Disorders

Susceptibility to ASD is determined by an interplay of genetic and environmental influences. Researchers discovered that contact with PM_2.5_ during the prenatal and postnatal periods increases susceptibility of a child toward ASD [40,69].

A case–control study in Southwestern Pennsylvania revealed ~50% rise in the risk of ASD when exposed to an average cumulative level of PM_2.5_ from 3 months before conception through the child’s second year (*p* = 0.046) [40]. In addition, a significant association was identified between the increased risk of ASD and maternal exposure to aircraft ultrafine emission (PM_0.1_) throughout pregnancy in a cohort study involving 370,723 singleton pregnancies (HR: 1.02, [95% CI: 1.01–1.03] per interquartile range [IQR] = 0.02 μg/m^3^ rise) [43]. In a nested case–control study, it was observed that the relation between ASD and PM_2.5_ exposure was more pronounced in the final trimester (OR = 1.42 per IQR raise in PM_2.5_; 95% CI: 1.09, 1.86) when adjusting for mutual factors [41]. Yet another population-based cohort study identified that the sensitive periods of PM_2.5_ exposure linked to ASD occurred early in pregnancy, demonstrating statistical significance over 1–27 weeks of gestation (cumulative HR = 1.14 [95% CI: 1.06, 1.23] per IQR [7.4 μg/m^3^] increase). Furthermore, sex-stratified analysis revealed that the connections between early gestational exposure and ASD were more pronounced in boys than girls (boys HR = 1.16 [95% CI: 1.08, 1.26]; girls HR = 1.06 [95% CI: 0.89, 1.26]) [42]. While these findings contribute crucial evidence regarding the potential negative impacts of PM_2.5_ exposure on the nervous system during pregnancy, the studies lack longitudinal tracking research on children’s long-term neurodevelopmental outcomes. They also fail to adequately identify the critical developmental stages of PM_2.5_ exposure and they overlook the moderating role of gender in its effects. 

### 6.2. Experimental Research

#### 6.2.1. Neural Damage and Brain Injury

PM_2.5_ exposure induces neural damage and brain injury [70]. A study found gestational CAP exposure can produce neuropathological changes [71], including microglial activation and ventricular enlargement, enlarged corpus callosum, decreased hippocampal volume, aberrant white matter development, and hypomyelination [72]. Additionally, most of these effects were either exclusive to boys or more pronounced in them. Exposure to elevated levels of TSP and PM_2.5_ led to the shortened length of DA neurons within the clusters of the raphe nuclei, decreased dopamine levels, and dyskinesia emergence [47]. Furthermore, exposure to PM_2.5_ solely triggered intracerebral hemorrhage and disrupted the maturation of neurovascular networks. Additionally, gestational PM_2.5_ exposure resulted in tau pathology in the cortical region of male descendants, which is essential for modulating neuronal maturation [73]. Previous studies have also reported that PM_2.5_ triggered seasonally influenced neuronal apoptosis and synaptic impairments [74].

#### 6.2.2. Impaired Learning and Memory

Following exposure to PM_2.5_, both sexes exhibited compromised learning and short-term memory abilities accompanied by the sustained activation of glial cells within the prefrontal cortex and corpus callosum [75]. A study provided evidence that early developmental exposure to low levels of ultrafine particles can result in enduring effects on the central nervous system (CNS) functions. These consequences include enduring deficits in cognitive abilities such as learning and short-term memory, alterations in impulsive conduct and motor skills, and some persistent changes in the neurochemical processes of the brain, especially in the prefrontal cortex, the area crucial for cognitive functions [75]. Moreover, there was proof suggesting that gestational PM_2.5_ exposure disrupted Hoxa5-mediated neuronal morphogenesis in male mice, resulting in impaired spatial learning and memory. Organic constituents such as PAHs exerted a more detrimental effect compared with the inorganic components [76].

#### 6.2.3. ASD

Data from several studies have suggested that exposure to PM from traffic during pregnancy and breastfeeding contributes to the incidence of ASD and cognitive deficits [77]. Early exposure to PM was found to engender persistent behavioral impairments, which vary based on sex and may be a potential contributing factor to neurodevelopmental disorders such as ASD [78]. A study noted that mice subjected to fine PM throughout the stages of their gestational and early neonatal periods decreased social engagement in both sexes, while male descendants demonstrated more extensive repetitive impairments [78]. Mice exposed to PM_2.5_ during pregnancy and the postnatal stages exhibited a more pronounced impact on the neuroinflammatory response; postnatal exposure of mice to ambient ultrafine particles reproduced numerous features consistent with ASD, such as repetitive and impulsive behaviors [79].

#### 6.2.4. Anxiety, Depression, and Fear

Prenatal exposure plays a pivotal role in engendering anxiety-like behavior observed in adulthood [80]. A study revealed that exposing mice to PM_0.1_ before mating and during gestation resulted in an increase in depressive-like reactions in male offspring [81]. Increased exposure to PM_2.5_ was strongly linked with heightened fearful behaviors in boys and girls at 6 months of age, and increased prenatal exposure to PM exhibited time-dependent implications that varied across sex [82]. Overall, these studies highlighted the importance of sex differences in related toxicology studies. Additional exploration is warranted to gain a comprehensive understanding of the specific mechanisms responsible for inducing aberrant development or inflicting damage upon the neurological system during the developmental period.

## 7. Mechanistic Considerations of PM_2.5_-Induced Developmental Toxicity

**Figure 1:**
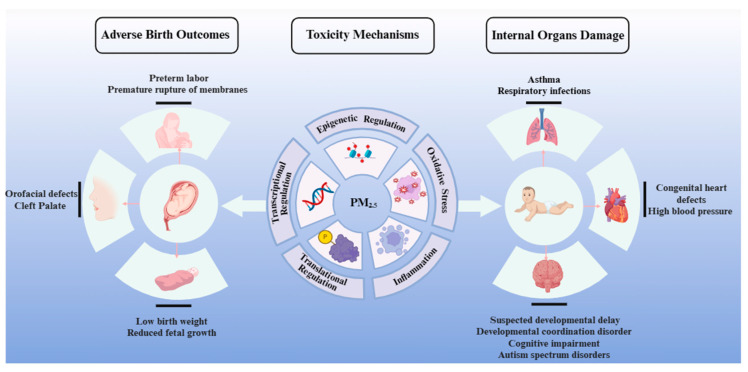
Schematic of biological mechanisms underlying PM_2.5_-induced adverse birth outcomes and internal organ developmental injury. Image created with BioRender.com accessed on 28 March 2024. with permission.

Air pollution, especially that caused by PM_2.5_, has attracted considerable attention as a prominent worldwide issue over the past several years, resulting in a substantial societal burden and loss of life. Extensive evidence from human and experimental research has revealed the deleterious impacts of PM, prompting a thorough investigation into the fundamental molecular processes (Figure 1). The negative health effects observed in offspring who were exposed to fine or ultrafine PM during their formative years are believed to be influenced by three primary mechanisms: transcriptional and translational regulation, oxidative stress and inflammation responses, and epigenetic regulation (developmental toxicity responses and mechanisms of PM_2.5_ are presented in Table 2). These mechanisms have offered insightful information regarding the connections between exposure to PM_2.5_ and health consequences, potentially aiding in the identification of targeted interventions that may be advantageous for pregnant women who are exposed to air pollutants.

### 7.1. Transcriptional and Translational Regulation

As external pollutants can affect gene expression and alter the synthesis, activity, and function of proteins in organisms, either through direct or indirect means, transcriptional and translation regulation are considered the primary regulatory mechanisms for the negative impacts of PM_2.5_. For instance, PM_2.5_ interferes with embryonic development by impacting processes such as autophagy and apoptosis, leading to cellular dysfunction often associated with alterations in the relevant genetic expression. Additionally, various receptor proteins, including components of the MAPK and caspases, have been involved in mediating these developmental toxic effects. A study provided evidence that encountering PM_2.5_ during gestation reduced the levels of autophagy-related proteins and suppressed autophagy flux primarily on GD15. Similarly, the activation of the AMPK/mTOR signaling pathway, which plays a crucial role in regulating autophagy, was also observed in the placenta on GD15. These findings revealed that the AMPK/mTOR pathway could be implicated in the inhibition of placental autophagy caused by PM_2.5_, leading to placental dysfunction and IUGR in mice [45]. Researchers attempted to address the potential involvement of ROS–MAPK–apoptosis/cell cycle arrest pathways in the embryotoxicity triggered by PM_2.5_, using whole rat embryos cultured in vitro. The results revealed that PM_2.5_ treatment delayed embryonic development along with cellular apoptosis and G0/G1 phase arrest.

Additionally, ROS production and the consequent activation of JNK and ERK signaling pathways may participate in these detrimental consequences by decreasing the Bcl-2/Bax protein ratio and increasing transcription levels of p15^INK4B^, p16^INK4A^, and p21^WAF1/CIP1^ [83,90]. Another study explored the correlation between PM_2.5_ exposure and neurodegenerative effects such as neuronal apoptosis and synaptic damage. The findings demonstrated that exposure to PM_2.5_ induced modifications in the expression of proteins associated with apoptosis, primarily bax and bcl-2, leading to the activation of caspase-3 and subsequent neuronal apoptosis. Moreover, PM_2.5_ exposure resulted in a reduction in the expression extent of synaptic structural postsynaptic density protein 95 (PSD-95) and synaptic functional protein N-Methyl-D-Aspartate (NMDA) receptor subunit (NR2B), both crucial for synaptic integrity and function. The suppression of phosphorylated ERK1/2 and CREB accompanied these effects. One additional key finding derived from this study was that these outcomes showed a seasonal correlation, with the most pronounced alterations observed in the PM_2.5_ samples obtained over the winter period [74].

The nervous system of zebrafish larvae experienced developmental toxicity due to exposure to PM_2.5_, which disrupted neuronal differentiation and neurovasculogenesis processes. Exposure to elevated concentrations of PM_2.5_ resulted in decreased expression levels of genes associated with dopamine regulation as well as essential neurodevelopmental genes linked to CNS development in zebrafish. This exposure also caused the loss and degeneration of dopaminergic neurons as well as decreasing the length of DA neurons in the raphe nuclei clusters [47]. This study presented empirical support for the adverse effects on cardiovascular health and neurodevelopment caused by acute exposure to TSP and PM_2.5_, which were linked to abnormal gene expression. Another study also concluded from a murine model that the prenatal inhalation of PM_2.5_ could potentially have detrimental consequences on the neurobehavior of offspring. This impact was linked to the overactivation of the dopamine pathway and inhibition of the glycine pathway within the brain [84].

A study examined the genetic expression profile of the cortical regions of male descendants following PM_2.5_ exposure during pregnancy. This experiment revealed that prenatal PM_2.5_ exposure disrupted myelin development via the lncRNAs–Ctcf signaling pathway, inducing abnormal myelin development in male offspring. The observation of reduced myelin sheath thickness in the optic nerves and a slight decline in myelin content in the corpus callosum serve as compelling evidence for this phenomenon. This study presented genomic evidence linking prenatal encounters with PM_2.5_ with neurodevelopmental disorders in male descendants [72].

In addition, researchers observed that the TGF-β/Smad3 pathway served as a mediator for the increased expression of EMT, which contributed to postnatal pulmonary impairment linked to maternal contact with PM_2.5_ in a rat model [52]. Additional research has demonstrated that exposure to PM_2.5_ during the gestational period contributes to cardiac impairment in newborns, primarily by modifying the protein expression associated with the regulation of Ca^2+^ [59]. 

To summarize, the studies mentioned above have delved deeply into the mechanisms through which PM_2.5_ regulates gene expression, elucidating that PM_2.5_ exerts direct effects on gene expression and protein function through multiple pathways such as interfering with the binding of transcription factors to DNA, regulating RNA stability, and influencing post-translational modifications. This impact not only induces changes at the cellular level but may also trigger cascading effects in crucial biological processes such as neurodevelopment and immune regulation. These findings broadened the comprehension of a range of transcriptional and translational regulation prompted by exposure to PM_2.5_, providing new insights for interventions and treatments of environmentally related diseases.

### 7.2. Oxidative Stress and Inflammation Response

It is widely accepted that oxidative stress and inflammation exert a crucial influence on the negative impacts of PM_2.5_ on the development process of respiratory, cardiovascular, and nervous systems. There are abundant published studies describing the contribution of oxidative stress and inflammation on various body systems.

Intrauterine exposure to PM_2.5_ raises susceptibility to acute infections of the bronchopulmonary system during the early developmental stage. Exposure to PM_2.5_ during pregnancy leads to the onset of lung inflammation in descendants through pathways involving elevated HMGB1 expression, which in turn facilitates the secretion of IL-1, IL-6, and TNF-α [51]. Upon inhalation, on the one hand, PM directly damages cells by generating free radicals; on the other hand, it can be detected by receptors present on macrophages in the respiratory system, both of which stimulate the release of inflammatory cytokines [91,92], induce inflammatory responses, and result in newborns and young infants being more vulnerable to pulmonary infections [12]. PM_0.1_ also penetrates the pulmonary endothelium and enters the bloodstream, reaching other organs [91,92]. This leads to an increase in systemic superoxide radical production due to subsequent neutrophil activation, which disrupts the integrity of endothelial cells [93]. Furthermore, a study found that higher exposure to PM_2.5_ was associated with increasing concentrations of MCP-1 and IL-6 chemokines in the body as well as an increased influx of macrophages and neutrophils into multiple organs, including adipose tissue, the lungs, spleen, and thymus [94].

While the precise mechanisms remain elusive, oxidative stress and inflammation are considered to have significant implications in heart development. One study provided evidence that PM_2.5_ caused abnormal heart development through oxidative stress, evidenced by altered Ca^2+^ regulatory proteins and increased levels of oxidative stress markers. Apart from oxidative stress, inflammatory response is also involved in this damage, supported by the observation that CRP expression increased nearly eightfold in offspring who were prenatally exposed to PM_2.5_ [57]. Another study indicated that EOM from PM_2.5_ increased mtROS production through the overexpression of CYP1A1 mediated by AHR, which aggravated the opening of the mPTP, resulting in mitochondrial dysfunction. The opening of the mPTP further caused the buildup of mtROS, ultimately leading to inherent apoptosis and heart defects. These findings indicate that mitochondria have the potential to be an attractive candidate for therapeutic targeting to prevent and treat CHDs caused by air pollution [85].

Similarly, EOM exposure triggered AHR/ROS-mediated ER stress, which subsequently intensified ROS production. Sustained ER stress upregulated CHOP, promoting DNA apoptosis and inhibiting Wnt signaling, consequently contributing to cardiac abnormalities in the embryos of the zebrafish species. This study revealed the participation of ER stress in the development of adverse cardiac defects caused by PM_2.5_, indicating that targeting ER stress and inhibiting CHOP upregulation could potentially treat and prevent CHDs caused by air pollution [64,66,67].

Air pollution poses a significant threat to the brain, and PM_2.5_ exposure during pregnancy might adversely impact the neurobehavior of descendants through an inflammatory response [79]. Possible mechanisms by which PM_2.5_ induces neurological damage include direct neurotoxicity caused by the permeation of PM into CNS tissues, triggering a cascade of secondary neurotoxic events that include reactive microglia proliferation triggered by inflammatory signaling molecules entering the brain from the circulation. Reportedly, ultrafine PM present in the bloodstream can traverse the blood–brain barrier and the cribriform plate in the olfactory mucosa, thereby entering the cerebrum [95]. Once within the cerebrum, PM may activate reactive microgliosis and interfere with the growth of neurons even at minimal levels while inducing the death of neurons at greater doses [75,79,95,96]. Another study reported that when pregnant individuals were exposed to UFP, their offspring exhibited the enduring activation of glial cells in the corpus callosum and frontal cortex [75]. This glial activation increased the generation of inflammatory cytokines such as IL-6 and TNF-α, which perpetuated the cycle of neuroinflammation and oxidative damage [97,98]. Exaggerated microglial activation and accompanying inflammation have the potential to cause hypomyelination and ventriculomegaly by exerting toxicity on oligodendrocytes, which are responsible for myelination in the cerebrum, ultimately resulting in impaired learning and short-term memory outcomes in both sexes [79,86,99,100,101,102,103]. Although there is much research on the inflammation and oxidative stress caused by PM_2.5_, mysteries still surround the detailed signaling pathways and interactions among various cellular factors. The relationship between the inflammatory and oxidative stress effects of different components of PM_2.5_ remains unclear. 

### 7.3. Epigenetic Regulation

Epigenetic regulation, encompassing DNA methylation, histone modification, and RNA-mediated processes, is critical in the developmental process. Epigenetic changes are responsive to external stressors and have been proposed to serve as a connection between environmental influences and genetic factors [104]. Early-life exposure to air pollutants might contribute to epigenetic dysregulation, causing developmental abnormalities, childhood and adult illnesses, and even the development of cancer later in life. To date, the molecular pathways underlying the mechanism by which PM_2.5_ induces epigenetic modifications have been inadequately explored.

DNA methylation is a constituent of the extensively researched epigenetic alterations and is essential in the development of the heart. Disruptions in DNA methylation have been implicated in the occurrence of CHD [105]. Extensive research has provided insights into the aberrant DNA methylation alterations triggered by PM_2.5_ exposure, which can result in disruptions of DNA methylation and mRNA expression, leading to abnormal embryonic development. Elevated prenatal exposure was correlated with an increased overall mutation rate in the placenta, which coincided with epigenetic modifications in crucial DNA repair and tumor suppressor genes, ultimately resulting in modifications in the capacity of fetal and neonatal DNA repair [106]. The transcriptome and DNA methyl group of hESCs were significantly influenced by the presence of EOM extracted from PM_2.5_. The integrated examination of alterations in DNA methylation and mRNA expression revealed an increased abundance of terms associated with the VEGFR signaling pathway and extracellular matrix. These findings offered a new understanding of the molecular processes that are implicated in the detrimental impact on development triggered by PM_2.5_ [88].

In addition, EOM exposure can engender significant modifications in DNA methylation either through modifying the activity of genes associated with DNA methylation or through reducing the SAM/SAH ratio. These disrupted DNA methylation patterns affect the transcription of genes implicated in cardiac development, leading to the manifestation of cardiac abnormalities in zebrafish embryos. A crucial discovery of this study is that the administration of folic acid supplements can counteract this sequence of adverse alterations induced by EOM, and the reduction in DNA methylation changes might be involved in the protective effect of folic acid against cardiac developmental toxicity [87]. m^6^A RNA methylation, which is the most prevalent type of RNA modification, has been identified as a critical factor in cardiac developmental toxicity.

One study indicated that exposure to EOM derived from PM_2.5_ leads to significant alterations in m^6^A RNA methylation. This is achieved through the downregulation of mettl14, facilitated by the AHR. Consequently, there is an upregulation of traf4a and bbc3 genes, triggering oxidative stress and apoptosis and eventually causing irregularities in cardiac development. Fortunately, this effect can be mitigated by the use of AHR antagonists and dietary methyl donors [89]. The collected data strongly support the fact that PM_2.5_ exposure leads to substantial epigenetic modifications, which may affect the activation of pivotal genes implicated in early developmental processes. These discoveries greatly enhanced the comprehension of how PM_2.5_ exerts its harmful effects at the molecular level and underscore the importance of studying the components of PM_2.5_. 

## 8. Conclusions

Although there is a growing body of information regarding the effects of exposure to PM during pregnancy, the complex composition and diverse sources of PM_2.5_, as well as the influence of environmental factors such as atmospheric stability and topographical features and the existence of differences in exposure levels and individual sensitivity, make it difficult to ascertain its potential health risks to humans and wildlife. Additionally, epidemiological studies may be affected by factors such as sample selection bias, information bias, or confounding, which could impact the accuracy and reliability of research results. In laboratory experiments, important factors such as species differences, exposure methods, duration, dosage, and particle composition may not be adequately discussed and considered, which could affect the scientific validity and credibility of the research conclusions. Therefore, the following aspects should be emphasized: (1) given the high adsorption capacity of PM_2.5_ for various pollutants, different origins of PM_2.5_ may lead to diverse negative impacts. Subsequent research should prioritize examining adducts generated by a range of contaminants, encompassing heavy metals and organic compounds while considering their combined effects and identifying the specific roles of various air toxins through the multipollutant modeling or an effect-directed analysis strategy; (2) long-term assessment, identification of critical exposure windows, and multiple influences such as mother’s age, economic level, and educational attainment should be considered; (3) the role of sexes on repercussions of atmospheric contamination deserves more research. Several studies have reported that there are differences in susceptibility to PM_2.5_ between sexes, and there may be a reciprocal relationship between genetic and external elements; and (4) the mechanism of PMs precrossing and postcrossing the placental barrier also needs further in-depth study, which can provide theoretical support for the treatment of PM-induced diseases.

In conclusion, there remain significant knowledge deficiencies in the comprehension of the complex signaling pathways that mediate cellular reactions to PM_2.5_. Advancing our comprehension regarding the mechanisms by which PM_2.5_ affects health will be instrumental in the creation of clinically relevant drugs and novel strategies to protect the public, especially children, from air pollution.

## Figures and Tables

**Table 2:** Summary of developmental toxicity responses and mechanisms of PM_2.5_ in vivo and in vitro.

Experimental Animals/Cells	Study Design	Biological Response	Molecular Mechanism	References
Mice fetal	Pregnant mice were randomized into concentrated PM_2.5_ group and FA group	Reduced fetal weight, crown–rump length, placental disorder and IUGR	AMPK/mTOR pathway	Li et al., 2022 [45]
SD rat offspring embryos	PM_2.5_ was added at 6.25–200 μg/mL	Impaired embryonic growth, decreased yolk sac size, crown–rump length, head length, and somite count	ROS-MAPKs-apoptosis/cell cycle arrest pathways	Yuan et al., 2016 [83]
Zebrafish embryos/larvae	Exposed to 25, 50, 100, 200, and 400 μg/mL of PM_2.5_ and TSP from 24 to 120 hpf	Elevated mortality rate, malformations, reduced development of cardiovasculature and neurovasculature	ERS and Wnt signaling	Jia et al., 2022 [47]
Male mice offspring	Pregnant mice were orally given PM_2.5_ suspension (3 mg/kg/2 days) until the birth of decedents	Impaired myelin ultrastructure on PNDs 14 and 21	lncRNAs–Ctcf signaling pathway	Hou et al., 2023 [72]
Primary cortical neuron	Treated with the PM_2.5_ samples at different concentration	Neuronal apoptosis and synaptic injuries	Suppression of phosphorylated ERK1/2 and CREB, activated caspase-3	Chen et al., 2017 [74]
Murine offspring	Pregnant ICR mice were exposed daily to PM_2.5_ (0.4 mg/m^3^) or FA, separately (for 14 consecutive days)	Locomotor hyperactivities	The excessive activity of the dopamine pathway inhibited the glycine pathway	Cui et al., 2019 [84]
Neonatal mice (14 days old)	Pregnant FVB female mice were exposed either to FA or PM_2.5_ at an average concentration of 91.78 μg/m^3^ for 6 h/day, 5 days/week throughout the gestation period (20 days)	Functional cardiac changes that were evident during the very early (14 days) stages of adolescence	Altered Ca^2+^ handling protein expression	Tanwar et al., 2017 [59]
Pups mice PND1 and 28	Timed pregnant SD rats were treated with PM_2.5_ (0.1, 0.5, 2.5, or 7.5 mg/kg) once every 3 days from day 0 to 18 of pregnancy	Significant decreases in lung volume parameters, compliance, and airflow during expiration on PND28, interstitial proliferation in lung histology	TGF-β/Smad3 pathway	Tang et al., 2017 [52]
Juvenile male rats	Gestational and early-life exposure to traffic-related PM (PM_2.5_ was 200 μg/m^3^) for 5 h/day, 5 days/week for 6 weeks	Decreased social behavior, increased anxiety, impaired cognition, disrupted neural integrity	Decreased levels of inflammatory and growth factors	Nephew et al., 2020 [77]
Mice offspring	Timed pregnant SD rats were treated with PM_2.5_ (0.1, 0.5, 2.5, or 7.5 mg/kg) once every 3 days from day 0 to 18 of pregnancy	Fetal lung injury, lung inflammation	Promoted IL-1, IL-6, and TNF-α secretion	Tang et al., 2018 [51]
Zebrafish embryo	Exposed to EOM concentration at 5 mg/L abstracted from PM_2.5_	Mitochondrial dysfunction, apoptosis and heart defects	CYP1A1 overexpression and accumulation of mtROS	Chen et al., 2023 [85]
Adult mice offspring	Male and female FVB mice were exposed to either FA or PM_2.5_ at an average concentration of 38.58 μg/m^3^ for 6 h/day, 5 days/week for 3 months	Cardiac dysfunction	Altered Ca^2+^ regulatory proteins, increased oxidative stress markers, inflammatory and fibrogenic mediators	Tanwar et al., 2018 [57]
Zebrafish embryos	Exposed to EOM abstracted from PM_2.5_ at different concentrations at 3 hpf in the absence or presence of CH (0.5 μM) or CHIR (1 μM)	Heart defects	Activation of AHR, repressed Wnt/β-catenin signaling	Zhang et al., 2016 [64]
Zebrafish embryos	Exposed to EOM (5 mg/L) in the absence or presence of CH (0.05 µM) or NAC (0.25 µM) from 3 hpf until 72 hpf	DNA damage and apoptosis, cardiac developmental toxicity	Oxidative stress	Ren et al., 2020 [66]
Zebrafish embryos	Treated with EOM (5 mg/L) in the absence or presence of 4-PBA (5 mM), CH (0.05 mM) or NAC (0.25 mM) from 2 to 72 hpf	Apoptosis, heart defects	ER stress and Wnt signaling inhibition	Zhang et al., 2022 [67]
Mice	Exposed in the postnatal period from PNDs 4–7 and 10–13, with adult re-exposure at PNDs 57–59	Long-term impairment in learning/short-term memory, impulsivity-linked behavior and motor function	Persistent glial activation, more inflammatory cytokines including IL-6, TNF-α	Allen et al., 2014 [75]
Mice	Exposed to concentrated ambient UFP from PND 4–7 and 10–13	Repetitive and impulsive behaviors, reductions in the size of the CC and associated hypomyelination	Inflammation/microglial activation, elevated glutamate and excitatory/inhibitory imbalance, increased amygdala astrocytic activation	Allen et al., 2017 [79]
Mice	Exposed to ultrafine CAPs or FA on PNDs 4–7 and 10–13	Lateral ventricle dilation	Neuroinflammatory response, alterations in cytokines and neurotransmitters	Allen et al., 2014 [86]
Zebrafish embryos	Exposed to EOM extracted from PM_2.5_ (5 mg/L) in the absence or presence of FA (0.05 μM) from 3 hpf	Heart defects	Decreased SAM/SAH ratio, altered expression of genes related to DNA methylation	Jiang et al., 2019 [87]
hESCs line H1	Treated with EOM from PM_2.5_ concentrations exceeding 100 μg/mL	Abnormal embryonic development, decrease in vitality	Interference with DNA methylation and mRNA expression	Wang et al., 2023 [88]
Zebrafish embryos	Treated with EOM concentrations of 5 mg/L	Apoptosis and cardiac malformations	Decreased global m^6^A RNA methylation levels	Ji et al., 2023 [89]

Abbreviations: AHR, aryl-hydrocarbon receptor; CC, corpus callosum; CH, CH223191; CHIR, CHIR99021; ERS, endoplasmic reticulum stress; FA, filtered air; FVB, friend leukemia virus b; hESCs, human embryonic stem cells; hpf, hours post-fertilization; IUGR, intrauterine growth restriction; PNDs, postnatal days; UFP, ultrafine particle (<100 nm).

## Data Availability

Not applicable.

## Abbreviations

AHR, aryl-hydrocarbon receptor; aOR, adjusted OR; ASD, autism spectrum disorders; ASQ, Ages and Stages Questionnaire; BC, black carbon; BP, blood pressure; CAP, concentrated ambient PM_2.5_; CC, corpus callosum; CH, CH223191; CHDs, congenital heart diseases; CHIR, CHIR99021; CNS, Central Nervous System; CP, cleft palate alone; DCD, developmental coordination disorder; EMT, epithelial–mesenchymal transition; EOM, extractable organic matters; ERS, endoplasmic reticulum stress; FA, filtered air; FVB, friend leukemia virus b; GD, gestation day; hESCs, human embryonic stem cells; hpf, hours post-fertilization; id, intellectual disability; IQ, intelligence quotient; IUGR, intrauterine growth restriction; LBW, low birth weight; LDCDQ, Little DCD Questionnaire; MDI, Mental Developmental Index; NMDA, N-Methyl-D-Aspartate; NR2B, receptor subunit; PDI, Psychomotor Developmental Index; PM_0.1_, ultrafine emission; PM_2.5_, particulate matter of size ≤ 2.5 μm; PNDs, postnatal days; PROM, premature rupture of membranes; PSD-95, postsynaptic density protein 95; PTB, preterm birth; SBP, systolic blood pressure; SDDs, suspected developmental delay; UFP, ultrafine particle (<100 nm).
